# Do we need antibiotic prophylaxis in endoscopic inguinal hernia repair? Results of the Herniamed Registry

**DOI:** 10.1007/s00464-015-4149-2

**Published:** 2015-03-19

**Authors:** F. Köckerling, R. Bittner, D. Jacob, C. Schug-Pass, C. Laurenz, D. Adolf, T. Keller, B. Stechemesser

**Affiliations:** 1 Department of Surgery and Center for Minimally Invasive Surgery, Academic Teaching Hospital of Charité Medical School, Vivantes Hospital, Neue Bergstraße 6, 13585 Berlin, Germany; 2 Winghofer Medicum, Hernia Center, Winghofer Straße 42, 72108 Rottenburg am Neckar, Germany; 3 StatConsult GmbH, Halberstädter Straße 40 a, 39112 Magdeburg, Germany; 4 Hernia Center Cologne, PAN – Hospital, Zeppelinstraße 1, 50667 Cologne, Germany

**Keywords:** Antibiotic prophylaxis, Endoscopic inguinal hernia repair, TAPP, TEP, Postoperative complications, Wound infection

## Abstract

**Introduction:**

The use of antibiotic prophylaxis in inguinal hernia repair is a controversial issue. Accepted randomized controlled trials or registry data with specific analysis of endoscopic repaired patients do not exist.

**Patient and methods:**

The data presented in this study compared the prospectively collected data from the Herniamed Registry on all patients who had undergone unilateral, bilateral or recurrent repair of inguinal hernias using either endoscopic or open techniques between September 1, 2009, and March 5, 2014. In total, 85,033 patients were enrolled. Of these patients, 48,201 (56.7 %) had an endoscopic and 36,832 (43.3 %) an open repair. The target variables analyzed were impaired wound healing and deep infections with mesh involvement within 30 days after the operation.

**Results:**

Analysis of the patient group with endoscopic/laparoscopic inguinal hernia repair (*n* = 48,201) did not identify any significant influence of antibiotic prophylaxis on postoperative impaired wound healing, which occurred in 53 cases (*p* = 0.6431). Nor was it possible to identify any significant impact of antibiotic prophylaxis on the deep infections seen in 27 cases (*p* = 0.8409). Analysis of the open inguinal hernia repair group revealed that, unlike the laparoscopic/endoscopic group, antibiotic prophylaxis had a significant impact on the postoperative impaired wound healing and deep infection rates. The risk of postoperative impaired wound healing with antibiotic prophylaxis was significantly lower [OR 0.677 (0.479; 0.958), *p* = 0.027].

**Conclusion:**

The positive impact of the endoscopic/laparoscopic technique on avoidance of impaired wound healing and deep infections with mesh involvement is already so great that antibiotic prophylaxis has no additional benefit. In contrast, antibiotic prophylaxis should be administered for open inguinal hernia repair.

The use of antibiotic prophylaxis in inguinal hernia surgical repair is a controversial issue. Prospective randomized controlled trials (RCTs) have identified postoperative infection rates (impaired wound healing, mesh infections) of between 0 and 8.9 % in the absence of antibiotic prophylaxis and of between 0 and 8.8 % on administration of antibiotic prophylaxis [1].

Of the 14 RCTs that compared inguinal hernia repair while using antibiotic prophylaxis versus placebo, 13 surgical procedures were performed using open repair and only one with an endoscopic technique [1].

That single RCT involving endoscopic inguinal hernia repair has incorrect randomization, lacks a definition of wound infection and is heavily underpowered with only 40 patients in each arm. It does not allow any conclusion to be made and is not included in the Cochrane review [1].

In a large case series with 8050 TAPP operations carried out for 6479 patients who had received antibiotic prophylaxis, the wound infection rate was 0 % and the mesh infection rate 0.1 % [2].

In a large case series with 5203 TEP operations involving 3868 patients who had received antibiotic prophylaxis, the wound infection rate was 0.08 % and the mesh infection rate 0.02 % [3].

The Swedish National Inguinal Hernia Registry recorded wound infection and mesh infection rates between 1992 and 2006. The incidence was 1.4 % in 28,220 patients registered as having received antibiotic prophylaxis. The infection rate was also 1.4 % in the non-prophylactic group, consisting of 104,354 patients. There was no specific analysis of the laparoscopic patients representing approximately 8 % of patients [1].

We now analyze below data from the Herniamed Registry to explore the influence that the use of an endoscopic technique for repair of inguinal hernia had on the rate of impaired wound healing and on deep infections with mesh involvement compared with open surgery. We will then also endeavor to elucidate whether administration of antibiotic prophylaxis further reduced the impaired wound healing and mesh infection rates.

## Patients and methods

The Herniamed quality assurance study is a multicenter, internet-based hernia registry into which 383 participating hospitals and surgeons in private practice in Germany, Austria and Switzerland (status: March 5, 2014) had entered data prospectively on their patients who had undergone hernia surgery [5].

The analysis now presented here compared the prospectively collected data on all patients who had undergone unilateral, bilateral or recurrent repair of inguinal hernia using either endoscopic or open techniques between September 1, 2009, and March 5, 2014. Inclusion criteria were minimum age of 16 years, primary or recurrent inguinal hernia and elective or emergency unilateral or bilateral inguinal hernia repair. In total, 85,033 patients were enrolled. Of these patients, 48,201 (56.7 %) had an endoscopic and 36,832 (43.3 %) an open repair. The data on these endoscopic inguinal hernia operations recorded in the Herniamed Registry originated from 315 out of 383 participating institutions.

The target variables analyzed were impaired wound healing and deep infections with mesh involvement within 30 days after the operation. The potential influence variables include, apart from the surgical technique and antibiotic prophylaxis, the patient’s age (years), sex (m/f), ASA status (I–IV), primary operation versus recurrent repair, hernia defect size (EHS Grade I–III) and general risk factors (nicotine, COPD, diabetes, cortisone, immunosuppression, etc.).

All analyses were performed with the software SAS 9.2 (SAS Institute Inc., Cary, NY, USA) and intentionally calculated to a full significance level of 5 %, i.e., they were not corrected in respect of multiple tests, and each *p* value ≤0.05 represents a significant result.

Since these data refer to unilateral as well as to bilateral operations, some of the variables used for analysis are given for both operated sides, but their values may differ. To ensure the independence of the sample elements used for analysis, the variables were aggregated as follows: In the case of a hernia defect size, the larger of the two defects was chosen and a postoperative infectious complication was considered to be present if at least one of the two sides experienced such a complication.

For unadjusted analyses of antibiotic influence on categorical outcome parameters, Fisher’s exact test was used. In case of more than two categories, exact analyses were not possible. Here, the asymptotic Chi-square test was used. Unadjusted analyses of continuous normal distributed outcome variables were realized using the robust *t* test (Satterthwaite).

Mainly, influences of antibiotic treatment and further confounding effects of patient characteristics were simultaneously reviewed in multivariable analyses. Potentially influencing factors were as follows: operating method (endoscopic/conventional), age (years), sex (male/female), ASA score (I–IV), defect size (EHS I–III), risk factors (nicotine, COPD, diabetes mellitus, aorta aneurysm, immunosuppression, cortisone, impaired coagulation, ASS/Plavix, Marcumar) and primary operation (yes/no). Risk factors were dichotomized, i.e., ‘yes’ if at least one risk factor is positive and ‘no’ otherwise.

Estimates for odds ratio (OR) and the corresponding 95 % confidence interval were given. For age [years], the 10-year OR estimate was given.

Since the properties of endoscopic and conventional techniques are presumably different, further analyses are made in these subgroups.

## Results

Between September 1, 2009, and March 5, 2014, *n* = 85,033 inguinal hernia operations were recorded in the Herniamed Registry in accordance with the inclusion criteria. Antibiotic prophylaxis was administered in *n* = 60,831 cases (71.54 %) and was not in 24,202 cases (28.46 %). Of the 60,831 cases that had received antibiotic prophylaxis, *n* = 59,177 (97.27 %) received single-shot antibiotic prophylaxis, *n* = 253 (0.42 %) for 1 day, *n* = 607 (0.99 %) for 2–3 days, and *n* = 794 (1.31 %) received antibiotic treatment for more than 3 days. No details of the antibiotic used were recorded in the registry study. Based on the recommendations of the German Paul-Ehrlich Society, in general ampicillin with a beta-lactamase inhibitor or a group 1 or 2 cephalosporin was administered [6]. As regards the surgical techniques employed for the analyzed patients, in the endoscopic group this was TAPP in *n* = 29,775 of cases (35 %) and TEP in *n* = 18,426 of cases (21.7 %), which thus accounted for 56.7 % of endoscopic procedures. In the open inguinal hernia repair group, Lichtenstein operation was performed in *n* = 23,820 of cases (28.0 %), Shouldice operation in *n* = 3057 (3.6 %), plug and patch technique in *n* = 3623 (4.3 %), Gilbert technique in *n* = 2554 (3.0 %) and TIPP technique in *n* = 1675 (2.0 %). Other techniques were used for *n* = 2103 (2.4 %).

### Unadjusted analyses

Analysis of the unadjusted correlation between antibiotic prophylaxis and the patient characteristics’ and operating technique variables demonstrated that there were highly significant differences across all variables between the patients who had and had not received antibiotic prophylaxis (in each case *p* < 0.001). For example, more laparoscopic/endoscopic operations were conducted while using antibiotic prophylaxis. Besides, the proportion of patients with risk factors and recurrences who had received antibiotic prophylaxis was significantly higher. The same was true for male gender and ASA classification. For low ASA status, antibiotic prophylaxis was rarely administered, with that applying also for smaller hernia defect sizes. Finally, patients who received antibiotic prophylaxis were on average just below 3 years older (Table 1).

**Table 1:** Demographic and surgery-related parameters

	Antibiotic prophylaxis	No antibiotic prophylaxis	*p* value
*Demographic parameters*			
Age			
Years ± SD	58.3 ± 16.3	55.6 ± 16.8	<0.001
Sex			
Male	53,703 (88.28 %)	21,141 (87.35 %)	
Female	7128 (11.72 %)	3061 (12.65 %)	<0.001
ASA score			
I	19,188 (31.54 %)	9,276 (38.33 %)	
II	31,028 (51.01 %)	12,111 (50.04 %)	
III	10,300 (16.93 %)	2740 (11.32 %)	
IV	315 (0.52 %)	75 (0.31 %)	<0.001
*Surgery-related parameters*			
Operation technique			
Laparoscopic	35,567 (58.47 %)	12,634 (52.20 %)	
Open	25,264 (41.53 %)	11,568 (47.80 %)	<0.001
Risk factors			
Yes	19,183 (31.53 %)	6373 (26.33 %)	
No	41,648 (68.47 %)	17,829 (73.67 %)	<0.001
Defect size			
I (<1.5 cm)	9772 (16.06 %)	4509 (18.63 %)	
II (1.5–3 cm)	35,381 (58.16 %)	13,383 (55.30 %)	
III (>3 cm)	15,678 (25.77 %)	6310 (26.07 %)	<0.001
Primary operation			
Yes	54,018 (88.80 %)	21,833 (90.21 %)	
No	6813 (11.20 %)	2369 (9.79 %)	<0.001

Unadjusted analysis of the outcome variables revealed that in the patient group that had received antibiotic prophylaxis, significantly fewer cases of impaired wound healing occurred compared with the patient group that had not received antibiotic prophylaxis (0.20 vs 0.30 %; *p* *=* 0 0.009). Likewise, on using antibiotic prophylaxis, there were significantly fewer cases of deep infections with mesh involvement (0.12 vs 0.20 %; *p* = 0.006) (Table 2).

**Table 2:** Postoperative complication rates of impaired wound healing and deep infection

Unadjusted analysis	Antibiotic prophylaxis	No antibiotic prophylaxis	*p* value
*Postoperative complications*			
Impaired wound healing			
Yes	123 (0.20 %)	73 (0.30 %)	
No	60,708 (99.80 %)	24,129 (99.70 %)	0.009
Deep infection			
Yes	71 (0.12 %)	48 (0.20 %)	
No	60,760 (99.88 %)	24,154 (99.80 %)	0.006

### Multivariable analysis of the total patient collective

The postoperative impaired wound healing rates were significantly influenced by antibiotic prophylaxis, surgical technique, sex, ASA classification and primary operations/recurrent repairs. Here antibiotic prophylaxis had a preventive effect on impaired wound healing [OR 0.706 (0.525; 0.949), *p* = 0.021], as did conduct of laparoscopic/endoscopic operation [OR 0.318 (0.230; 0.439), *p* < 0.001]. Likewise, there were significantly fewer cases of postoperative impaired wound healing among men [OR 0.531 (0.369; 0.764), *p* < 0.001] as well as for primary hernias [OR 0.601 (0.412; 0.876), *p* = 0.008]. The negative influence of the ASA status was imputed especially to the increased impaired wound healing rates observed for ASA IV status [IV vs I: OR 4.226 (1.579; 11.311) *p* = 0.001] (Table 3).

**Table 3:** Multivariable analysis of impaired wound healing in all patients with open and endoscopic inguinal hernia repair

Parameter	*p*	Categories	OR estimate	95 % CI	
				Lower CL	Upper CL
Operation technique	<0.001	Laparoscopic versus open	0.318	0.230	0.439
ASA score	<0.001	II versus I	0.750	0.524	1.073
		III versus I	1.301	0.795	2.129
		IV versus I	4.226	1.579	11.311
Sex	<0.001	Male versus female	0.531	0.369	0.764
Primary operation	0.008	Yes versus no	0.601	0.412	0.876
Antibiotic prophylaxis	0.021	Yes versus no	0.706	0.525	0.949
Defect size	0.107	I (<1.5 cm) versus III (> 3 cm)	0.618	0.387	0.984
		II (1.5–3 cm) versus III (>3 cm)	0.789	0.574	1.085
Age (10-year OR)	0.331		0.951	0.859	1.052
Risk factors	0.707	Yes versus no	0.939	0.675	1.305

Postoperative deep infection with mesh involvement was affected by antibiotic prophylaxis, surgical technique and ASA classification. An OR of 0.593 (0.408; 0.862), *p* = 0.006, was calculated for antibiotic prophylaxis. The risk of postoperative deep infection was significantly lower for the laparoscopic/endoscopic surgical technique [OR 0.259 (0.167; 0.402), *p* < 0.001]. Deep infections also occurred significantly more often among ASA IV patients [IV vs I: OR 5.425 (1.732; 16.992), *p* = 0.008] (Table 4).

**Table 4:** Multivariable analysis of deep infection in all patients with open and endoscopic inguinal hernia repair

Parameter	*p*	Categories	OR estimate	95 % CI	
				Lower CL	Upper CL
Operation technique	<0.001	Laparoscopic versus open	0.259	0.167	0.402
Antibiotic prophylaxis	0.006	Yes versus no	0.593	0.408	0.862
ASA score	0.008	II versus I	0.946	0.584	1.532
		III versus I	1.541	0.814	2.916
		IV versus I	5.425	1.732	16.992
Defect size	0.229	I (<1.5 cm) versus III (>3 cm)	1.189	0.697	2.029
		II (1.5–3 cm) versus III (>3 cm)	0.795	0.524	1.206
Risk factors	0.278	Yes versus no	1.250	0.835	1.873
Sex	0.582	Male versus female	0.865	0.515	1.451
Primary operation	0.756	Yes versus no	0.917	0.532	1.582
Age (10-year OR)	0.871		1.011	0.886	1.154

In summary, it can be concluded that impaired wound healing and deep infections occurred significantly less often after endoscopic/laparoscopic inguinal hernia surgery compared with open repair. In the total patient collective comprising *n* = 48,201 endoscopic/laparoscopic and *n* = 36,832 open inguinal hernia operations, antibiotic prophylaxis significantly reduced impaired wound healing and deep infection rates.

### Multivariable analysis of laparoscopic/endoscopic inguinal hernia operations

Analysis of the patient group with endoscopic/laparoscopic inguinal hernia repair (*n* = 48,201) did not identify any significant influence exerted by the different variables on postoperative impaired wound healing, which occurred in 53 cases (*p* = 0.6431). Nor was it possible to identify any significant impact of the variables on the deep infections seen in 27 cases (*p* = 0.8409) (Table 5).

**Table 5:** Multivariable analysis of impaired wound healing and deep infection in all patients with endoscopic inguinal hernia repair

		Impaired wound healing	Deep infection
Model fitting			
(Global test)	*p*	0.6431	0.8409

### Multivariable analysis of open inguinal hernia operations

Analysis of the open inguinal hernia repair group revealed that, unlike the laparoscopic/endoscopic group, antibiotic prophylaxis had a significant impact on the postoperative impaired wound healing and deep infection rates. The risk of postoperative impaired wound healing with antibiotic prophylaxis was significantly lower [OR 0.677 (0.479; 0.958), *p* = 0.027] (Table 6). Given a total rate of 0.39 %, this would correspond to 31 cases of impaired wound healing for every 10,000 open repair operations performed under antibiotic prophylaxis and to 46 such complications for every 10,000 open repairs carried out without antibiotic prophylaxis. Likewise, the postoperative deep infection rate was found to be significantly reduced with antibiotic prophylaxis [OR 0.522 (0.341; 0.798), *p* = 0.003] (Table 7). Accordingly, for every 10,000 open repair operations and a total rate of 0.25 %, 17 postoperative infections would occur under antibiotic prophylaxis compared with 33 without antibiotic administration.

**Table 6:** Multivariable analysis of impaired wound healing in all patients with open inguinal hernia repair

Parameter	*p*	Categories	OR estimate	95 % CI	
				Lower CL	Upper CL
ASA score	<0.001	II versus I	0.795	0.515	1.227
		III versus I	1.485	0.837	2.634
		IV versus I	5.106	1.836	14.200
Primary operation	0.001	Yes versus no	0.512	0.339	0.774
Sex	0.003	Male versus female	0.532	0.350	0.807
Antibiotic prophylaxis	0.027	Yes versus no	0.677	0.479	0.958
Defect size	0.267	I (<1.5 cm) versus III (>3 cm)	0.646	0.377	1.109
		II (1.5–3 cm) versus III (>3 cm)	0.829	0.576	1.195
Age (10-year OR)	0.446		0.955	0.848	1.075
Risk factors	0.532	Yes versus no	0.886	0.605	1.296

**Table 7:** Multivariable analysis of deep infection in all patients with open inguinal hernia repair

Parameter	*p*	Categories	OR estimate	95 % CI	
				Lower CL	Upper CL
Antibiotic prophylaxis	0.003	Yes versus no	0.522	0.341	0.798
ASA score	0.010	II versus I	0.900	0.507	1.597
		III versus I	1.539	0.744	3.181
		IV versus I	5.241	1.593	17.246
Defect size	0.024	I (<1.5 cm) versus III (>3 cm)	1.218	0.688	2.157
		II (1.5–3 cm) versus III (>3 cm)	0.601	0.375	0.962
Risk factors	0.107	Yes versus no	1.454	0.922	2.292
Age (10-year OR)	0.580		1.044	0.897	1.216
Primary operation	0.589	Yes versus no	0.850	0.470	1.535
Sex	0.728	Male versus female	0.901	0.500	1.623

## Discussion

The present Herniamed Registry study investigated the influence exerted by antibiotic prophylaxis on the occurrence of infectious complications following laparoscopic/endoscopic inguinal hernia operations and compared these findings with those obtained for open inguinal hernia surgery. Besides, other risk factors were identified for onset of impaired wound healing and deep infections with mesh involvement. This also applies for patients with risk factors. For the total patient collective comprising 85,033 patients with 48,201 endoscopic/laparoscopic and 36,832 open inguinal hernia operations, it was revealed that postoperative infectious complications were significantly reduced by use of the endoscopic/laparoscopic technique and antibiotic prophylaxis. Indeed, analysis even revealed that the influence exerted by the endoscopic/laparoscopic technique on onset of postoperative infectious complications had a more preventive effect than that of antibiotic prophylaxis. Hence, the minimally invasive procedure for inguinal hernia repair made a greater contribution to the prevention of impaired wound healing and deep infections with mesh involvement than did antibiotic prophylaxis.

If one analyzes the group of 48,201 endoscopic/laparoscopic surgery patients as a separate entity, one notes that the use of antibiotic prophylaxis was unable to reduce further the rates of impaired wound healing and deep infections with mesh involvement. Hence, antibiotic prophylaxis should not be administered for endoscopic/laparoscopic inguinal hernia surgery. Conversely, analysis of the 36,832 open inguinal hernia repair group showed that antibiotic prophylaxis had a significant impact on onset of postoperative impaired wound healing and deep infections with mesh involvement. Accordingly, antibiotic prophylaxis should be administered for open inguinal hernia repair.

Of the 14 prospective randomized controlled trials that compared inguinal hernia surgery under antibiotic prophylaxis versus placebo, the endoscopic technique was performed only in one case trial [4]. However, due to methodological drawbacks that study was not taken into account in the Cochrane review [7].

Nor has the impact of antibiotic prophylaxis on occurrence of infectious complications following endoscopic/laparoscopic inguinal hernia operations been investigated by other studies recorded in the review [1]. As such, the Guidelines of the European Hernia Society and the International Endohernia Society do not contain any clear-cut recommendations as to whether antibiotic prophylaxis should or should not be administered for endoscopic/laparoscopic inguinal hernia repair [1, 8]. This present study by the Herniamed Registry has now clearly demonstrated for a large patient collective that antibiotic prophylaxis does not bestow additional benefits in the case of endoscopic/laparoscopic inguinal hernia surgery. The positive impact of the endoscopic/laparoscopic technique on avoidance of impaired wound healing and deep infections with mesh involvement is already so great that antibiotic prophylaxis has no additional benefit.

However, the converse situation applies for open inguinal hernia repair. Multivariable analysis identified that antibiotic prophylaxis had a significant effect on onset of impaired wound healing and deep infections with mesh involvement. That concords with the findings of several meta-analyses [9–12]. Conversely, the Cochrane review [7] and a systematic review and meta-analysis [13] concluded that antibiotic prophylaxis did not prevent the occurrence of wound infection after groin hernia surgery. In an update of Guidelines of the European Hernia Society [8], antibiotic prophylaxis is thus only recommended for high postoperative infection rates (>5 %). While the total rate of postoperative impaired wound healing and deep infections for open inguinal hernia surgery given in the Herniamed Registry is markedly lower than that, it has been possible to demonstrate the positive effect of antibiotic prophylaxis on the basis of a large patient collective. Accordingly, impaired wound healing would occur only in 31 rather than in 46 of every 10,000 cases. It would be possible to reduce almost by half the proportion of deep infections from 33 to 17 for every 10,000 operations. Therefore, antibiotic prophylaxis should be administered for open inguinal hernia surgical repair.

## Appendix: Herniamed study group

### Scientific board

**Köckerling**, Ferdinand (Chairman); **Berger**, Dieter; **Bittner**, Reinhard; **Fortelny**, René; **Koch**, Andreas; **Kraft,** Barbara; **Kuthe**, Andreas; **Lorenz**, Ralph; **Mayer**, Franz; **Moesta**, Kurt Thomas; **Niebuhr**, Henning; **Peiper**, Christian; **Pross**, Matthias; **Reinpold**, Wolfgang; **Simon**, Thomas; **Stechemesser**, Bernd; **Unger**, Solveig

### Participants

**Ahmetov**, Azat (Saint-Petersburg); **Alapatt,** Terence Francis (Frankfurt/Main); **Anders,** Stefan (Berlin); **Anderson**, Jürina (Würzburg); **Arndt**, Anatoli (Elmshorn); **Asperger,** Walter (Halle); **Avram**, Iulian (Saarbrücken); **Barkus**; Jörg (Velbert); **Becker**, Matthias (Freital); **Behrend**, Matthias (Deggendorf); **Beuleke,** Andrea (Burgwedel); **Berger,** Dieter (Baden-Baden); **Bittner,** Reinhard (Rottenburg); **Blumberg,** Claus (Lübeck); **Böckmann,** Ulrich (Papenburg); **Böhle**, Arnd Steffen (Bremen); **Böttger,** Thomas Carsten (Fürth); **Borchert,** Erika (Grevenbroich); **Born**, Henry (Leipzig); **Brabender,** Jan (Köln); **Breitenbuch von**, Philipp (Radebeul); **Brüggemann**, Armin (Kassel); **Brütting**, Alfred (Erlangen); **Budzier**, Eckhard (Meldorf); **Burghardt**, Jens (Rüdersdorf); **Carus**, Thomas (Bremen); **Cejnar**, Stephan-Alexander (München); **Chirikov**, Ruslan (Dorsten); **Comman,** Andreas (Bogen); **Crescent**i, Fabio (Verden/Aller); **Dapunt**, Emanuela (Bruneck); **Decker**, Georg (Berlin); **Demmel**, Michael (Arnsberg); **Descloux,** Alexandre (Baden); **Deusch**, Klaus-Peter (Wiesbaden); **Dick**, Marcus (Neumünster); **Dieterich**, Klaus (Ditzingen); **Dietz**, Harald (Landshut); **Dittmann**, Michael (Northeim); **Dornbusch**, Jan (Herzberg/Elster); **Drummer**, Bernhard (Forchheim); **Eckermann**, Oliver (Luckenwalde); **Eckhoff,** Jörn /Hamburg); **Elger**, Karlheinz (Germersheim); **Engelhardt**, Thomas (Erfurt); **Erichsen,** Axel (Friedrichshafen); **Eucker**, Dietmar (Bruderholz); **Fackeldey**, Volker (Kitzingen); **Farke**, Stefan (Delmenhorst); **Faust**, Hendrik (Emden); **Federmann**, Georg (Seehausen); **Feichter**, Albert (Wien); **Fiedler,** Michael (Eisenberg); **Fischer**, Ines (Wiener Neustadt); **Fortelny**, René H. (Wien); **Franczak**, Andreas (Wien); **Franke**, Claus (Düsseldorf); **Frankenberg von**, Moritz (Salem); **Frehner**, Wolfgang (Ottobeuren); **Friedhoff**, Klaus (Andernach); **Friedrich,** Jürgen (Essen); **Frings**, Wolfram (Bonn); **Fritsche**, Ralf (Darmstadt); **Frommhold,** Klaus (Coesfeld); **Frunder**, Albrecht (Tübingen); **Fuhrer**, Günther (Reutlingen); **Gassler,** Harald (Villach); **Gerdes**, Martin (Ostercappeln); **Gilg**, Kai-Uwe (Hartmannsdorf); **Glaubitz**, Martin (Neumünster); **Glutig,** Holger (Meißen); **Gmeiner**, Dietmar (Bad Dürrnberg); **Göring**, Herbert (München); **Grebe**, Werner (Rheda-Wiedenbrück); **Grothe**, Dirk (Melle); **Gürtler**, Thomas (Zürich); **Hache**, Helmer (Löbau); **Hämmerle**, Alexander (Bad Pyrmont); **Haffner**, Eugen (Hamm); **Hain**, Hans-Jürgen (Groß-Umstadt); **Hammans**, Sebastian (Lingen); **Hampe**, Carsten (Garbsen); **Harrer**, Petra (Starnberg); **Heinzmann**, Bernd (Magdeburg); **Heitland**, Tim (München); **Helbling**, Christian (Rapperswil); **Hempen**, Hans-Günther (Cloppenburg); **Henneking**, Klaus-Wilhelm (Bayreuth); **Hermes**, Wolfgang (Weyhe); **Herrgesell**, Holger (Berlin); **Herzing**, Holger Höchstadt); **Hessler**, Christian (Bingen); **Hildebrand**, Christiaan (Langenfeld); **Höferlin**, Andreas (Mainz); **Hoffmann**, Michael (Kassel; **Hofmann**, Eva M. (Frankfurt/Main); **Hopfe**r, Frank (Eggenfelden); **Hornung**, Frederic (Wolfratshausen); **Hügel**, Omar (Hannover); **Hüttemann**, Martin (Oberhausen); **Huhn**, Ulla (Berlin); **Imdahl**, Andreas (Heidenheim); **Jacob**, Dietmar (Bielefeld); **Jenert**, Burghard (Lichtenstein); **Jugenheimer**, Michael (Herrenberg); **Junger**, Marc (München); **Käs**, Stephan (Weiden); **Kahraman,** Orhan (Hamburg); **Kaiser,** Christian (Westerstede); **Kaiser**, Stefan (Kleinmachnow); **Kapischke**, Matthias (Hamburg); **Karch**, Matthias (Eichstätt); **Keck**, Heinrich (Wolfenbüttel); **Keller,** Hans W. (Bonn); **Kienzle**, Ulrich (Karlsruhe); **Kipfmüller**, Brigitte (Köthen); **Kirsch**, Ulrike (Oranienburg); **Klammer**, Frank (Ahlen); **Klatt**, Richard (Hagen); **Kleemann**, Nils (Perleberg); **Klein**, Karl-Hermann (Burbach); **Kleist**, Sven (Berlin); **Klobusicky**, Pavol (Bad Kissingen); **Kneifel**, Thomas (Datteln); **Knoop**, Michael (Frankfurt/Oder); **Knotter**, Bianca (Mannheim); **Koch,** Andreas (Cottbus); **Köckerling,** Ferdinand (Berlin); **Köhler**, Gernot (Linz); **König**, Oliver (Buchholz); **Kornblum**, Hans (Tübingen); **Krämer**, Dirk (Bad Zwischenahn); **Kraft**, Barbara (Stuttgart); **Kreissl**, Peter (Ebersberg); **Krones**, Carsten Johannes (Aachen); **Kruse,** Christinan (Aschaffenburg); **Kube**, Rainer (Cottbus); **Kühlberg**, Thomas (Berlin); **Kuhn,** Roger (Gifhorn); **Kusch,** Eduard (Gütersloh); **Kuthe,** Andreas (Hannover); **Ladberg**, Ralf (Bremen); **Ladra**, Jürgen (Düren); **Lahr-Eigen**, Rolf (Potsdam); **Lainka**, Martin (Wattenscheid); **Lammers**, Bernhard J. (Neuss); **Lancee**, Steffen (Alsfeld); **Larusson**, Hannes Jon (Pinneberg); **Lauschke**, Holger (Duisburg); **Leher,** Markus (Schärding); **Leidl**, Stefan (Waidhofen/Ybbs); **Lenz**, Stefan (Berlin); **Lesch**, Alexander (Kamp-Lintfort); **Lienert**, Mark (Duisburg); **Limberger**, Andreas (Schrobenhausen); **Locher**, Martin (Kiel); **Loghmanieh**, Siawasch (Viersen); **Lorenz**, Ralph (Berlin); **Mallmann**, Bernhard (Krefeld); **Manger**, Regina (Schwabmünchen); **Maurer**, Stephan (Münster); **Mayer**, Franz (Salzburg); **Menzel**, Ingo (Weimar); **Meurer**, Kirsten (Bochum); **Meyer**, Moritz (Ahaus**)**; **Mirow**, Lutz (Kirchberg); **Mittenzwey**, Hans-Joachim (Berlin); **Mörder-Köttgen**, Anja (Freiburg); **Moesta**, Kurt Thomas (Hannover); Moldenhauer, Ingolf (Braunschweig); **Morkramer**, Rolf (Xanten); **Mosa**, Tawfik (Merseburg); **Müller**, Hannes (Schlanders); **Münzberg**, Gregor (Berlin); **Mussack,** Thomas (St. Gallen); **Neumann**, Jürgen (Haan); **Niebuhr,** Henning (Hamburg); **Nölling**, Anke (Burbach); **Nostitz**, Friedrich Zoltán (Mühlhausen); **Obermaier**, Straubing); **Öz-Schmidt**, Meryem (Hanau); **Oldorf**, Peter (Usingen); **Olivieri**, Manuel (Pforzheim); **Pawelzik**, Marek (Hamburg); **Peiper**, Christian (Hamm); **Pertl**, Alexander (Spittal/Drau); **Philipp**, Mark (Rostock); **Pickart**, Lutz (Bad Langensalza); **Pizzera**, Christian (Graz); **Pöllath**, Martin (Sulzbach-Rosenberg); **Possin**, Ulrich (Laatzen); **Prenzel**, Klaus (Bad Neuenahr-Ahrweiler); **Pröve**, Florian (Goslar); **Pronnet**, Thomas (Fürstenfeldbruck); **Pross**, Matthias (Berlin); **Puff**, Johannes (Dinkelsbühl); **Rabl**, Anton (Passau); **Rapp**, Martin (Neunkirchen); **Reck**, Thomas (Püttlingen); **Reinpold,** Wolfgang (Hamburg); **Reuter**, Christoph (Quakenbrück); **Richter,** Jörg (Winnenden); **Riemann**, Kerstin (Alzenau-Wasserlos); **Rodehorst**, Anette (Otterndorf); **Roehr**, Thomas (Rödental); **Roncossek**, Bremerhaven); **Roth** Hartmut (Nürnberg); **Sardoschau**, Nihad (Saarbrücken); **Sauer**, Gottfried (Rüsselsheim); **Sauer**, Jörg (Arnsberg); **Seekamp**, Axel (Freiburg); **Seelig**, Matthias (Bad Soden); **Seiler**, Christoph Michael (Warendorf); **Seltmann,** Cornelia (Hachenburg); **Senkal,** Metin (Witten); **Shamiyeh**, Andreas (Linz); **Shang**, Edward (München); **Siemssen**, Björn (Berlin); **Sievers,** Dörte (Hamburg); **Silbernik**, Daniel (Bonn); **Simon**, Thomas (Sinsheim); **Sinn**, Daniel (Olpe); **Sinning**, Frank (Nürnberg); **Smaxwil**, Constatin Aurel (Stuttgart); **Schabel**, Volker (Kirchheim/Teck); **Schadd**, Peter (Euskirchen); **Schassen von**, Christian (Hamburg); **Schattenhofer**, Thomas (Vilshofen); **Scheidbach**, Hubert (Neustadt/Saale); **Schelp**, Lothar (Wuppertal); **Scherf**, Alexander (Pforzheim); **Scheyer**, Mathias (Bludenz); **Schimmelpenning,** Hendrik (Neustadt in Holstein); **Schinkel**, Svenja (Kempten); **Schmid**, Michael (Gera); **Schmid,** Thomas (Innsbruck); **Schmidt**, Rainer (Paderborn); **Schmidt**, Sven-Christian (Berlin); **Schmidt,** Ulf (Mechernich); **Schmitz**, Heiner (Jena); **Schmitz**, Ronald (Altenburg); **Schöche**, Jan (Borna); **Schoenen**, Detlef (Schwandorf); **Schrittwieser**, Rudolf /Bruck an der Mur); **Schroll**, Andreas (München); **Schultz**, Christian (Bremen-Lesum); **Schultz**, Harald (Landstuhl); **Schulze**, Frank P. Mülheim an der Ruhr); **Schumacher**, Franz-Josef (Oberhausen); **Schwab**, Robert (Koblenz); **Schwandner**, Thilo (Lich); **Schwarz**, Jochen Günter (Rottenburg); **Schymatzek**, Ulrich (Radevormwald); **Spangenberger**, Wolfgang (Bergisch-Gladbach); **Sperling**, Peter (Montabaur); **Staade**, Katja (Düsseldorf); **Staib**, Ludger (Esslingen); **Stamm**, Ingrid (Heppenheim); **Stark**, Wolfgang (Roth); **Stechemesser,** Bernd (Köln); **Steinhilper**, Uz (München); **Stern**, Oliver (Hamburg); **Stolte**, Thomas (Mannheim); **Stopinski,** Jürgen (Schwalmstadt); **Stubbe**, Hendrik (Güstrow/); **Stülzebach**, Carsten (Friedrichroda); **Tepel,** Jürgen (Osnabrück); **Terzić**, Alexander (Wildeshausen); **Teske,** Ulrich (Essen); **Thews**, Andreas (Schönebeck); **Tillenburg**, Wolfgang (Marktheidenfeld); **Timmermann,** Wolfgang (Hagen); **Train**, Stefan H. (Gronau); **Trauzettel**, Uwe (Plettenberg); **Triechelt**, Uwe (Langenhagen); **Ulcar**, Heimo (Schwarzach im Pongau); **Unger**, Solveig (Chemnitz); **Verweel**, Rainer (Hürth); **Vogel**, Ulrike (Berlin); **Voigt**, Rigo (Altenburg); **Voit**, Gerhard (Fürth); **Volkers**, Hans-Uwe (Norden); **Vossough**, Alexander (Neuss); **Wallasch**, Andreas (Menden); **Wallner**, Axel (Lüdinghausen); **Warscher,** Manfred (Lienz); **Warwas**, Markus (Bonn); **Weber**, Jörg (Köln); **Weiß**, Johannes (Schwetzingen); **Weißenbach**, Peter (Neunkirchen); **Werner**, Uwe (Lübbecke-Rahden); **Wessel,** Ina (Duisburg); **Weyhe**, Dirk (Oldenburg); **Wieber**, Isabell (Köln); **Wiesmann**, Aloys (Rheine); **Wiesner**, Ingo (Halle); **Woehe**, Fritz (Sanderhausen); **Wolf**, Claudio (Neuwied); **Yildirim**, Selcuk (Berlin); **Zarras**, Konstantinos (Düsseldorf); **Zeller**, Johannes (Waldshut-Tiengen); **Zhorzel**, Sven (Agatharied); **Zuz**, Gerhard (Leipzig);

## References

[1] Bittner R, Arregui ME, Bisgaard T, Dudai M, Ferzli GS, Fitzgibbons RJ, Fortelny RH, Klinge U, Koeckerling F, Kuhry E, Kukleta J, Lomanto D, Misra MC, Montgomery A, Morales-Condes S, Reinpold W, Rosenberg J, Sauerland S, Schug-Pass C, Singh K, Timoney M, Weyhe D, Chowbey P (2011). Guidelines for laparoscopic (TAPP) and endoscopic (TEP) treatment of inguinal hernia [International Endohernia Society (IEHS)]. Surg Endosc.

[2] Bittner R, Schmedt CG, Schwarz J, Kraft K, Leible BJ (2002). Laparoscopic transperitoneal procedure for routine repair of groin hernia. Br J Surg.

[3] Tamme C, Scheidbach H, Hampe C, Schneider C, Köckerling F (2003). Totally extraperitoneal endoscopic inguinal hernia repair (TEP). Surg Endosc.

[4] Schwetling R, Bärlehner E (1998). Is there an indication for general perioperative antibiotic prophylaxis in laparoscopic plastic hernia repair with implantation of alloplastic tissue?. Zentralbl Chir.

[5] Stechemesser B, Jacob DA, Schug-Paß C, Köckerling F (2012). Herniamed: an internet-based registry for outcome research in hernia surgery. Hernia.

[6] Wacha H, Hyome U, Isenmann R, Kujath P, Lebert C, Naber K, Salzberger B (2010). Perioperative antibiotika-prophylaxe. Chemother J.

[7] Sanchez-Manuel FJ, Lozano-Garcia J, Seco-Gil JL (2012). Antibiotic prophylaxis for hernia repair. Cochrane Database Syst Rev.

[8] Miserez M, Peeters E, Aufenacker T, Bouillot JL, Campanelli G, Conze J, Fortelny R, Heikkinen T, Jorgensen LN, Kukleta J, Morales-Conde S, Nordin P, Schumpelck V, Smedberg S, Smietanski M, Weber G, Simons MP (2014). Update with level 1 studies of the European Hernia Society guidelines on the treatment of inguinal hernia in adult patients. Hernia.

[9] Sanabria A, Dominguez LC, Valdivieso E, Gómez G (2007). Prophylactic antibiotics for mesh inguinal hernioplasty. Ann Surg.

[10] Li JF, Lai DD, Zhang XD, Zhang AM, Sun KX, Luo HG, Yu Z (2012). Meta-analysis of the effectiveness of prophylactic antibiotics in the prevention of postoperative complications after tension-free hernioplasty. Can J Surg.

[11] Yin YY, Song T, Liao B, Luo Q, Zhou Z (2012). Antibiotic prophylaxis in patients undergoing open mesh repair of inguinal hernia: a meta-analysis. Am Surg.

[12] Mazaki T, Mado K, Masuda H, Shiono M (2013). Antibiotic prophylaxis for the prevention of surgical site infection after tension-free hernia repair: a Bayesian and frequentist meta-analysis. J Am Coll Surg.

[13] Aufenacker TJ, Koelemay MJW, Gouma DJ, Simons MP (2006). Systematic review and meta-analysis of the effectiveness of antibiotic prophylaxis in prevention of wound infection after mesh repair of abdominal wall hernia. Br J Surg.

